# Targeting early changes in the synovial microenvironment: a new class of immunomodulatory therapy?

**DOI:** 10.1136/annrheumdis-2018-214294

**Published:** 2018-12-14

**Authors:** Susan R Aungier, Alison J Cartwright, Anja Schwenzer, Jennifer L Marshall, Michael R Dyson, Peter Slavny, Kothai Parthiban, Aneesh Karatt-Vellatt, Ilfita Sahbudin, Eric Culbert, Patrick Hextall, Felix IL Clanchy, Richard Williams, Brian D Marsden, Karim Raza, Andrew Filer, Christopher Dominic Buckley, John McCafferty, Kim S Midwood

**Affiliations:** 1 Kennedy Institute of Rheumatology, Nuffield Department of Orthopaedics, Rheumatology and Musculoskeletal Sciences, University of Oxford, Oxford, UK; 2 Institute of Inflammation and Ageing, University of Birmingham, Queen Elizabeth Hospital, Birmingham, UK; 3 IONTAS Ltd, Cambridge, UK; 4 Nascient Ltd, Cambridge, UK; 5 Structural Genomics Consortium, Nuffield Department of Clinical Medicine, University of Oxford, Oxford, UK; 6 Department of Rheumatology, Sandwell and West Birmingham Hospitals NHS Trust, Birmingham, UK

**Keywords:** extracellular matrix, inflammation, tenascin-C, monoclonal antibodies, rheumatoid arthritis

## Abstract

**Objectives:**

Controlled immune responses rely on integrated crosstalk between cells and their microenvironment. We investigated whether targeting proinflammatory signals from the extracellular matrix that persist during pathological inflammation provides a viable strategy to treat rheumatoid arthritis (RA).

**Methods:**

Monoclonal antibodies recognising the fibrinogen-like globe (FBG) of tenascin-C were generated by phage display. Clones that neutralised FBG activation of toll-like receptor 4 (TLR4), without impacting pathogenic TLR4 activation, were epitope mapped by crystallography. Antibodies stained synovial biopsies of patients at different stages of RA development. Antibody efficacy in preventing RA synovial cell cytokine release, and in modulating collagen-induced arthritis in rats, was assessed.

**Results:**

Tenascin-C is expressed early in the development of RA, even before disease diagnosis, with higher levels in the joints of people with synovitis who eventually developed RA than in people whose synovitis spontaneously resolved. Anti-FBG antibodies inhibited cytokine release by RA synovial cells and prevented disease progression and tissue destruction during collagen-induced arthritis.

**Conclusions:**

Early changes in the synovial microenvironment contribute to RA progression; blocking proinflammatory signals from the matrix can ameliorate experimental arthritis. These data highlight a new drug class that could offer early, disease-specific immune modulation in RA, without engendering global immune suppression.

**Key messages**

**What is already known about this subject?**

- Immunomodulatory signals from the extracellular matrix help to shape immune responses. Activation of toll-like receptor 4 (TLR4) by tenascin-C, a matrix molecule persistently expressed at high levels in people with RA, drives chronic inflammation in models of rheumatoid arthritis (RA).

**What does this study add?**

- We developed monoclonal antibodies that block the TLR4 binding epitope within the fibrinogen-like globe domain of tenascin-C; these antibodies inhibit cytokine release by RA synovial cells and prevent disease progression and tissue destruction during collagen-induced arthritis.

**How might this impact clinical practice?**

- This study indicates that antibodies targeting proinflammatory signals from the extracellular matrix should be further explored for use in clinical practice for treating RA.

## Introduction

Environmental signals play a key role in shaping cell identity, imprinting tissue-specific gene expression programmes to enable geographically adapted cell behaviour. This includes, for example, specialisation of gut and brain macrophages, or of synovial and dermal fibroblasts, to fulfil distinct site-specific roles.^1 2^ Dynamic tissue remodelling during inflammation creates new microenvironmental niches designed to drive immune responses that restore homeostasis. These temporary structures comprise specialised extracellular matrix molecules that support infiltrating immune cells and proliferating tissue resident cells, pattern soluble effector molecules and signal to cells to orchestrate controlled inflammation.^3 4^ Immunomodulatory matrix molecules exhibit restricted expression in healthy tissue, but are persistently expressed at sites of pathological inflammation, leading to their exploitation in the clinic as disease-specific postcodes with which to deliver antibody-linked packages of cytotoxic and anti-inflammatory drugs.^5^ Here, we determined whether directly targeting the activity of these matrix molecules could combat pathological inflammation.

Tenascin-C is a large, multimodular extracellular matrix molecule that exhibits limited expression in healthy tissues but is transiently upregulated on cellular stress and tissue injury, where it triggers inflammation by activating toll-like receptor 4 (TLR4). Persistent expression of tenascin-C has been implicated as a driver of chronic inflammation in autoimmune, neurological, metabolic and fibrotic diseases, in which expression levels can predict prognosis and reflect treatment outcome.^6^ In patients with rheumatoid arthritis (RA), high tenascin-C is associated with more erosive joint disease and predicts poor response to biological treatment.^7^ During experimental joint disease, mice lacking tenascin-C are protected from prolonged synovial inflammation and tissue destruction; while inflammation is induced in these animals, it is also swiftly resolved, concomitant with downregulation of key inflammatory cytokines and pathogenic T cell subsets.^8 9^

Mapping the active domain within tenascin-C revealed a unique structural epitope in the fibrinogen-like globe (FBG) that is essential for binding to and activating TLR4.^8 10^ Distinct modes of receptor activation and diverse downstream signalling induced by FBG compared with pathogenic TLR4 agonists,^11^ revealed an opportunity to ablate pathological ‘sterile’ inflammation, leaving intact host defence against infection. We reasoned that this makes tenascin-C an attractive candidate for safely modulating inflammatory signals from the microenvironment. However, lack of specific, effective antagonists that block FBG activation of TLR4 have precluded assessment of tenascin-C as a viable therapeutic target.

## Methods

All methods are provided in the online supplementary materials section.

10.1136/annrheumdis-2018-214294.supp1 Supplementary data

## Results

### Generating neutralising anti-tenascin-C antibodies

**Figure 1:**
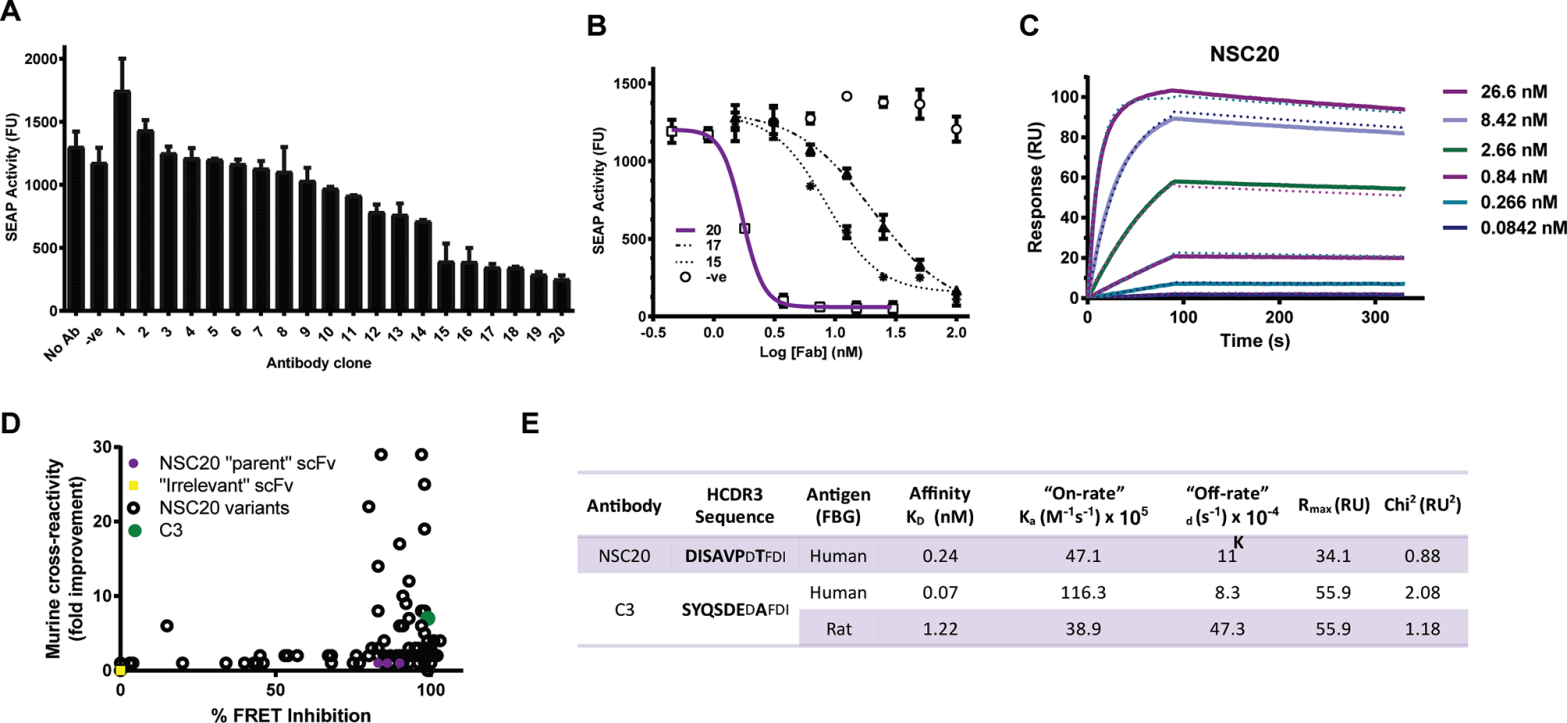
Generation and affinity maturation of anti-tenascin-C antibodies. (A) Fab clones were screened for their ability to inhibit FBG-mediated upregulation of nuclear factor kappa-light-chain-enhancer of activated B cells (NF-κB) in THP1-blue cells. (B) The potency of top hits (clones 15, 17 and 20) was further assessed compared with control Fab D1.3 recognising hen egg lysozyme. (A and B) Data are shown as the mean±SD from three experiments. (C) The binding affinity of NSC20 to human FBG was analysed by surface plasmon resonance (SPR) spectroscopy at 25°C. (D) Following affinity maturation, NSC20 variants were screened for improved binding to human and mouse FBG. HTRF measured the ability of affinity matured scFv clones to compete with labelled NCS20 IgG for antigen binding. Data are presented as percentage inhibition of the FRET signal (x-axis). The ENC assay used limiting amounts of immobilised anti-FLAG antibody to capture FLAG-tagged scFv from culture supernatants and thereby normalise the amount of immobilised scFv. After washing to remove excess (unbound) scFv, the ability of immobilised antibody clones to bind biotinylated CD4-his-mTNC-FBG was detected by DELFIA, allowing ranking of clones based on affinity. Parental NSC20 was used as a benchmark, and data are presented as fold-increase in fluorescence signal (y-axis). Open circles represent NSC20 variants, solid green circle indicates lead variant, C3. (E) The heavy chain CDR3 (HCDR3) sequence and binding properties, determined by SPR at 37°C, of C3 are shown compared with the parental antibody NSC20. In contrast to NSC20, no measurable dissociation of the antibody–antigen complex was detectable over a 10 min period at 25°C for C3 (data not shown). Hence, affinity values for C3 were determined at 37°C. ENC, expression-normalised capture; FBG, fibrinogen-like globe; HTRF, homogeneous time resolved fluorescence.

We generated monoclonal antibodies against the FBG domain of human tenascin-C using phage display. A panel of 20 sequence unique antibodies that bound to the FBG domain of tenascin-C, but not tenascin-R, the family member possessing the most closely related FBG domain, were selected for conversion into Fab format. Fabs were tested for blockade of nuclear factor kappa-light-chain-enhancer of activated B cells (NF-κB) activity induced in human monocytic THP1 reporter cells by stimulation with the FBG domain of tenascin-C (figure 1A). Titration of selected antibodies revealed a half maximal inhibitory concentration (IC_50_) of 1.7 nM for clone NSC20 (figure 1B). NSC20 bound to the FBG domain of human tenascin-C with high affinity (K_D_ 110 pM at 25°C) (figure 1C) and bound comparably well to canine FBG; however, binding to rodent FBG domains was 117-fold less (data not shown). To generate antibodies whose efficacy could be assessed in human and rodent models of disease, NSC20 was affinity matured. Among 138 individual NSC20 variants whose binding was analysed using homogeneous time resolved fluorescence competition and expression-normalised capture (ENC) assays (figure 1D), clone C3 exhibited a K_D_ for the FBG domain of human tenascin-C of 70 pM and recognised rat antigen with a K_D_ of 1.2 nM (figure 1E).

### Defining antibody mode of action

**Figure 2:**
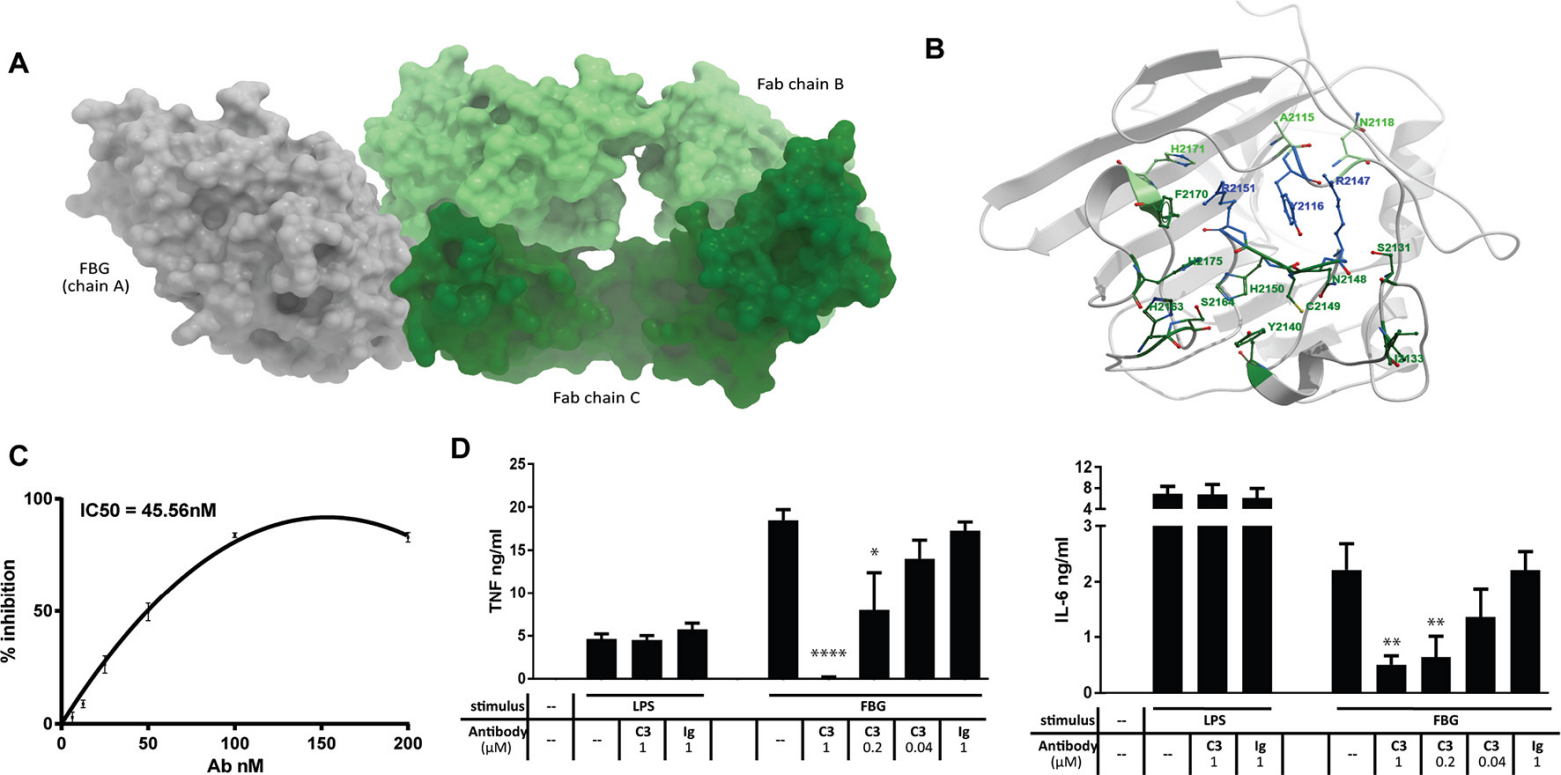
Defining the mode of action of neutralising anti-tenascin-C antibodies. (A and B) The crystal structure of C3 bound to the FBG domain of human tenascin-C. Six FBG residues bind to Fab chain B (heavy chain) (A2115, Y2116, R2147, R2151, N2118 and H2171) and 13 FBG residues with Fab chain C (light chain) (Y2116, S2131, I2133, Y2140, R2147, N2148, C2149, H2150, R2151, H2163, S2164, F2170 and H2175), three of which are shared (Y2116, R2147 and R2151). Light green: FBG residues in contact with Fab chain B; dark green: FBG residues in contact with Fab chain C; blue: FBG residues in contact with Fab chains B and C. This interaction interface is not conserved in the FBG domains of tenascin-R and tenascin–W. Only 9 of these 16 residues are present in FBG-R, and only seven in FBG-W. Of the three residues in FBG-C that interact with both heavy and light chain of the antibody, one residue is substituted in FBG-R, and all three are substituted in FBG-W. These data support experimental evidence that FBG-R does not bind to C3, and the higher sequence divergence of the FBG domain of tenascin-W with that of tenascin-C (54.1%) compared with tenascin-R (61.6%), and the fact that this divergence includes key positions in the C3 binding epitope, predicts that FBG-W will also be unable to bind to C3. (C) Recombinant human TLR4 was coated onto a 96-well plate, and recombinant human tenascin-C FBG, which had been preincubated with C3 or isotype control antibody, was added. Bound FBG was detected, and the percentage inhibition in the C3 preincubated samples was calculated compared with the isotype control samples (IC50=45.5 nM). Data are shown as the mean±SEM from eight experiments. (D) Recombinant human tenascin-C FBG (FBG) (1 µM) or lipopolysaccharide (LPS) (1 ng/mL) were preincubated with the indicated doses of C3 or isotype control antibody (Ig) before being added to primary human macrophages. After 24 hours, supernatants were taken, and cytokine ELISAs were performed. Data are shown as the mean±SEM from four independent donors. One-way analysis of variance was performed to determine significance of C3 inhibition compared with isotype control. *p<0.05, **p<0.01, ****p<0.0001. FBG, fibrinogen-like globe; IL, interleukin; TLR4, toll-like receptor 4; TNF, tumour necrosis factor.

Crystallisation of the FBG domain of human tenascin-C in complex with Fab fragments of C3 revealed interactions in the X-ray structure are mediated by hydrogen bonds and three layer pi:pi stacking, predominantly between one of the two Fab chains (figure 2A,B). Comparison of the TLR4 binding epitope^10^ with the Fab epitope in FBG predict that antibody binding will abrogate tenascin-C’s ability to activate TLR4 by preventing access of the receptor to residues (S2131 and I2133) in FBG that are required for optimal binding to TLR4. This was validated experimentally by demonstration that preincubation of FBG with C3 inhibits binding of FBG to purified recombinant TLR4 in a solid phase binding assay (IC_50_ 45.56 nM)(figure 2C) and that preincubation of FBG with C3 blocks the ability of FBG to induce cytokine synthesis in primary human macrophages, while C3 had no effect on LPS-induced cytokine release (figure 2D).

### Assessing antibody efficacy in synovial cells from patients with RA and in experimental arthritis

**Figure 3:**
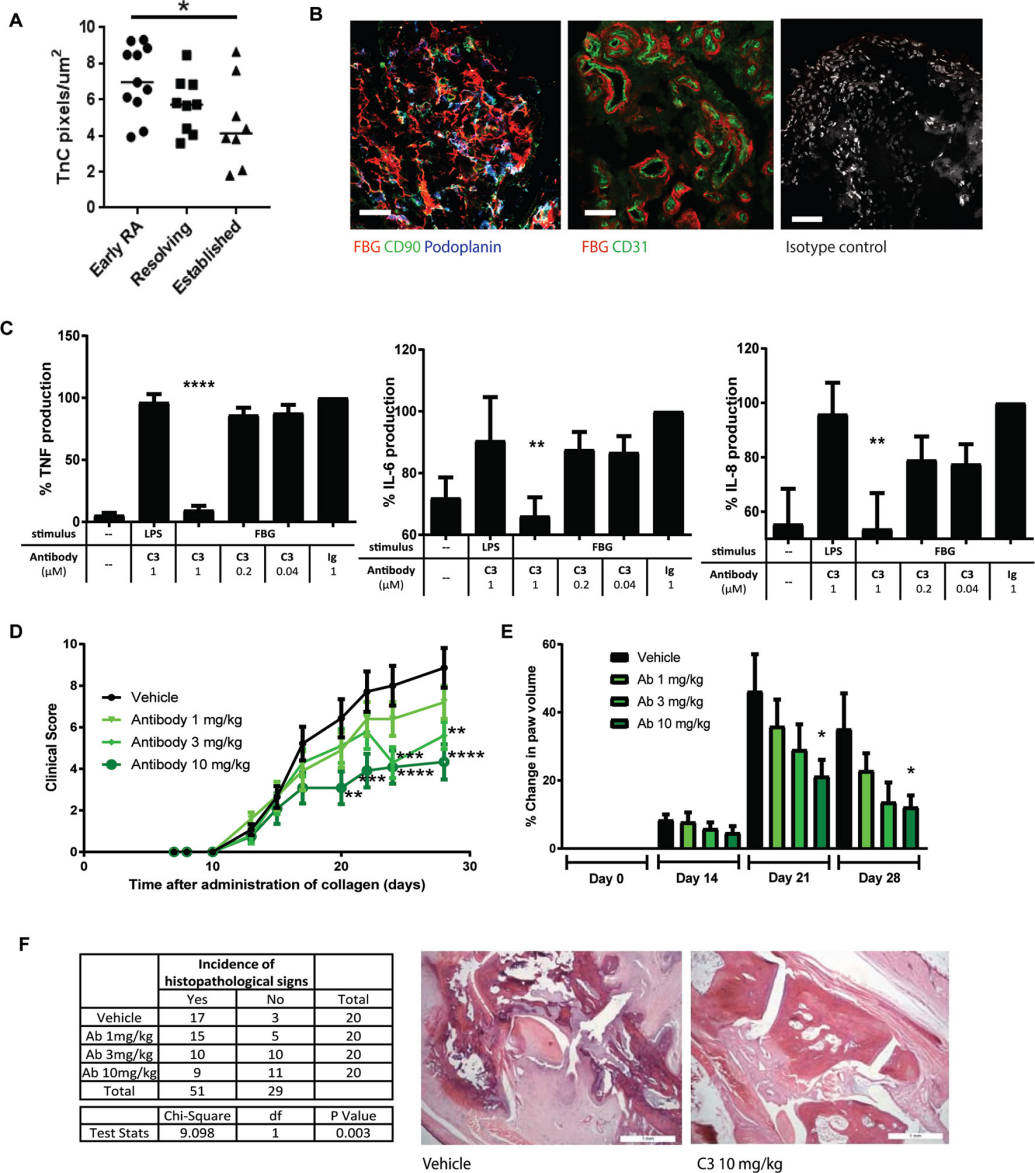
Antibody treatment ameliorates inflammation in experimental arthritis. (A and B) Synovial biopsies from the Birmingham early arthritis cohort were stained with anti-FBG antibodies. Quantification of the number of positive pixels per microgram biopsy was performed in tissue from people with early RA (undiagnosed synovitis of less than 3 months, patients who go on to be diagnosed with RA), people with synovial inflammation that spontaneously resolved (undiagnosed synovitis of less than 3 months that disappears by itself) (resolving) and people with established RA (diagnosed disease, greater than 3 months’ duration) (A). Anti-FBG staining was observed in areas of fibrosis in inflamed synovia (left panel: red anti-FBG, green anti-CD90, blue antipodoplanin) and around areas of vascularisation underneath endothelial cells (centre panel: red anti-FBG, green anti-CD31). No staining was observed with isotype controls in place of primary antibodies (right panel). Scale bars 50 µm (B). (C) Mixed populations of cells isolated from RA synovial membranes were stimulated with 1 µM recombinant human tenascin-C FBG or 1 ng/mL LPS, which had been preincubated with either C3 or isotype control antibody. After 24 hours, supernatants were taken and cytokine ELISAs were performed. Data are shown as the mean±SEM from three independent donors. One-way analysis of variance (ANOVA) was performed to determine significance of C3 inhibition compared with isotype control. *p=0.02, ***p<0.0001. (D–F) Rats were injected with bovine type II collagen intradermally on day 0 and day 7. Treatments of PBS (vehicle control) and C3 at 1, 3 or 10 mg/kg were administered twice weekly throughout the experiment by intravenous injection (10 animals per treatment group). Animals were scored for clinical signs of disease three times per week, and the mean±SEM is shown (D). Paw volumes were measured using a plethysmometer on days 0, 14, 21 and 28, and the mean change±SEM normalised to the day 0 measurements is shown (E). Two-way was carried out to test for significance of changes between vehicle and treatment groups. ****p<0.0001, ***p<0.001, **p<0.01, *p<0.05. (F) At termination on day 28, hind limbs were assessed for histological signs of inflammation, articular cartilage damage and damage to the underlying metaphyseal bone. A χ^2^ test for trend was used to confirm that the presence or absence of histopathological signs is associated with antibody treatment (χ^2^=9.098, p=0.003), with only 3 of 20 paws were free of any sign of histopathology in the vehicle treated group, whereas 11 of 20 paws were disease free in the 10 mg/kg treated group (table). Representative histological images of destructive arthritis (vehicle treated) and a normal joint (10 mg/kg) are shown (right panels). FBG, fibrinogen-like globe; IL, interleukin; RA, rheumatoid arthritis; TNF, tumour necrosis factor.

Staining with anti-FBG antibodies was observed in synovial biopsies from people with joint inflammation; tenascin-C levels were higher in people with early RA, compared with people who had joint inflammation that spontaneously resolved and who did not develop RA, or patients with established RA (figure 3A). FBG staining was predominantly observed in the sublining synovial layer of inflamed tissue where it created a dense matrix surrounding both podoplanin-positive and CD90-positive fibroblasts. FBG staining was also associated with blood vessels, lying underneath and around the CD31+ endothelial cell layer (figure 3B). Costaining of the C-terminal FBG domain with antibodies that recognise the N-terminal epidermal growth factor-like (EGF-L) repeats of tenascin-C revealed largely overlapping localisation and also highlighted areas where anti-FBG staining predominated (online supplementary figure 1). In mixed cell populations isolated from the synovium of patients with RA undergoing joint replacement, monoclonal antibody C3 blocked cytokine release induced by stimulation with FBG, but not LPS (figure 3C). Rats in which joint inflammation was induced by intradermal administration of type II collagen (day 0 and day 7) were treated twice weekly with vehicle (PBS), isotype control (10 mg/kg) or C3 (1, 3 or 10 mg/kg) from day 0 until the end of the experiment at day 28. No significant differences between vehicle and isotype control groups were observed for any parameter measured. However, increasing doses of C3 significantly reduced clinical score (figure 3D) and paw swelling (figure 3E). In addition, C3 treatment reduced the number of affected paws per animal; rats with only one affected paw were restricted to the 3 mg/kg and the 10 mg/kg C3 groups, and rats treated with 10 mg/kg C3 gained significantly more weight throughout the experiment (not shown). Finally, C3 treatment reduced the incidence of histopathological changes in the joint (χ^2^=9.098, p=0.003) (figure 3F).

## Discussion

This study describes the production of monoclonal antibodies that prevent the FBG domain of tenascin-C from binding to and activating TLR4. Staining biopsies of inflamed synovia with these antibodies revealed protein expression very early in RA and at higher levels than in people with established disease. Prophylactic administration of anti-FBG antibodies to rats with collagen-induced arthritis did not affect the induction of joint inflammation but inhibited disease progression and prevented joint damage. These data highlight that early changes in the synovial microenvironment contribute to the development of RA and that blocking inflammatory signals from the extracellular matrix could offer a new therapeutic strategy for treating this disease.

Development of anti-FBG antibodies provides evidence of a non-redundant role for tenascin-C activation of TLR4 in experimental models of joint inflammation. These antibodies also constitute a useful tool with which to learn more about how endogenous inflammatory stimuli shape immune responses. Both stromal and immune cells express TLR4 in the RA joint; identification of biological processes and effector molecules that are modulated by FBG blockade in each of these different cell types during disease amelioration may reveal new opportunities for suppressing inflammation. This will also inform preclinical benchmarking studies, for example, if anti-FBG treated animals phenocopy tenascin-C null animals, antibody treatment would block persistent synthesis of several cytokines from different cellular sources, including tumour necrosis factor, interleukin (IL)-6 and IL-17,^9^ raising the possibility that this approach could be more effective than single cytokine blockade.

Current approaches to targeting TLR4 in RA focus on antibodies that prevent receptor dimerisation, offering blockade of TLR4 activation by a broad range of pathogenic and endogenous ligands.^12 13^ These antibodies are well tolerated in healthy adults and are currently in phase 2a trials in patients with RA,^14^ for treatment of TLR4-driven disease defined by serum autoantibody signature.^15^ Here, we show that targeting a single endogenous TLR4 agonist is sufficient to offer therapeutic benefit in arthritis models. This strategy could enable a move away from blocking TLR4-mediated inflammation at the receptor level, hitting only disease-specific stimuli, without engendering global immune suppression. Tenascin-C is dispensable for the induction of joint inflammation but required for its persistence(figure 3),^8^ indicating that its blockade can be used simply to restore the resolution of inflammation, without hindering immune defence. This premise can now be interrogated by assessing the susceptibility of anti-FBG treated animals to infection, and by comparison of the efficacy and safety profiles of anti-FBG and anti-TLR4 antibodies. Reducing the risk of opportunistic or recurrent latent infection^16–19^ would be a significant step forward in the management of RA.

Treating RA early provides significant clinical benefit to patients.^20^ However, while a myriad of dysregulated signalling pathways and cytokine networks contribute to persistent inflammation in well-established disease, events that dictate progression from early synovitis to chronic joint inflammation and tissue destruction remain incompletely understood. This study reveals discreet, tenascin-C-rich niches around blood vessels and at sites of fibrosis in inflamed synovia, arising early in disease development. These data implicate changes in the synovial microenvironment in the onset of disease, warranting further investigation of the therapeutic window within which anti-FBG treatment can achieve efficacy and if this offers a realistic avenue for treating people with early disease.

Finally, while antibodies that specifically target FBG highlight this domain of tenascin-C as a critical driver of chronic synovial inflammation, the capacity of tenascin-C to exert both beneficial and deleterious effects across different joint tissues^21^ illustrates a fascinating context specificity for this molecule that remains poorly understood. For example, administration of exogeneous full-length tenascin-C prevents cartilage degeneration during murine models of osteoarthritis^22^ and promotes cartilage repair when applied to osteochondral defects in rabbits.^23^ It is not yet clear whether activation of TLR4 by the FBG domain occurs in isolation from, or in synergy with, signalling by other tenascin-C domains, how signals from this multidomain molecule are integrated within complex tissue networks and how tissue-specific responses to tenascin-C are mediated. While distribution of the EGF-L repeats and the FBG domain of tenascin-C overlap in inflamed synovia, areas of single antibody positivity may indicate locally elevated availability of the TLR4-activating epitope, for example, via tenascin-C conformations that expose the FBG domain and conceal the EGF-L repeats, or generation of FBG-containing proteolytic fragments. Better understanding how different forms of tenascin-C are distributed across different tissues, as well as in discreet niches within tissues, will provide further mechanistic insight into how this matrix molecule influences cell behaviour in situ.
