# Supplementary material for: Targeting early changes in the synovial microenvironment: a new class of immunomodulatory therapy?
Source: Ann Rheum Dis. 2018 Dec 14;78(2):186–91. doi: 10.1136/annrheumdis-2018-214294 (PMC6352652; doi:10.1136/annrheumdis-2018-214294)
Supplement: Supplementary data [file annrheumdis-2018-214294supp001.doc]

**SUPPLEMENTARY MATERIAL FOR**

**Targeting early changes in the synovial microenvironment:**

**a new class of immunomodulatory therapy?**

Susan R. Aungier^1^, Alison J. Cartwright^1^, Anja Schwenzer^1^, Jennifer L. Marshall^2^, Michael R. Dyson^3^, Peter Slavny^3^, Kothai Parthiban^3^, Aneesh Karatt-Vellatt^3^, Ilfita Sahbudin^2^, Eric Culbert^4^, Patrick Hextall^4^, Felix Clanchy^1^, Richard Williams^1^, Brian D. Marsden^1,5^, Karim Raza^2,6^, Andrew Filer^2^, Christopher D. Buckley^1,2^, John McCafferty^3^ and Kim S. Midwood^1*^


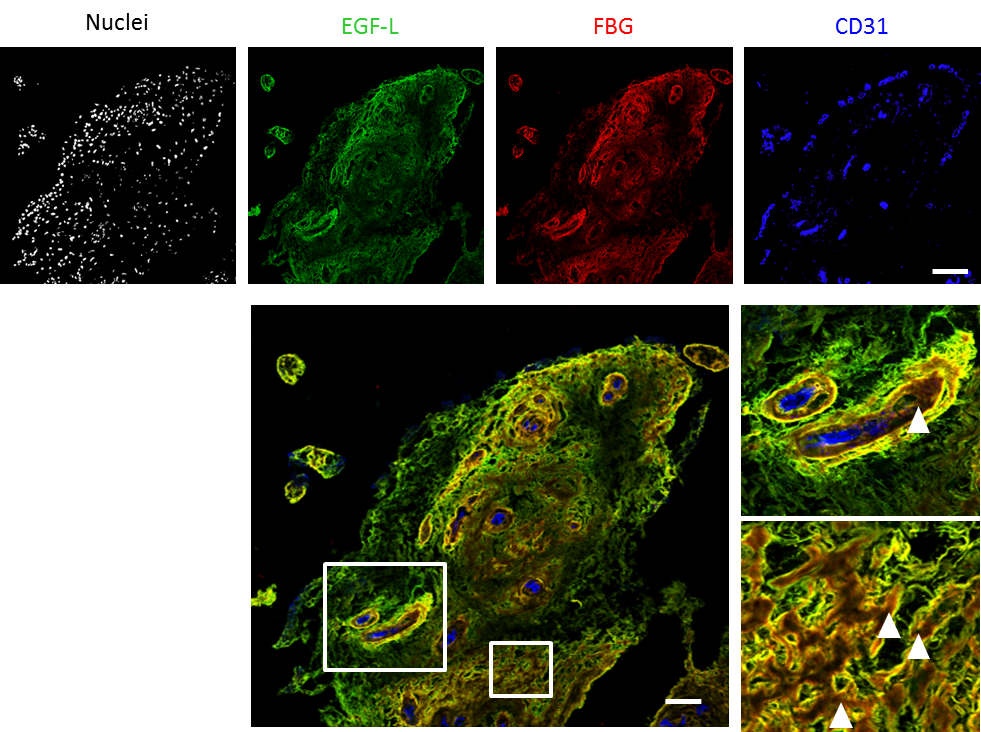
**Supplementary figure 1**

**Supplementary Figure 1. Comparison of the localization of the EGF-L and FBG domains of tenascin-C.** Synovial biopsies were co-stained with anti-EGF-L (green), anti-FBG (red) and anti-CD31 (blue) antibodies (single channel images: top right three panels). No staining was observed in serial sections incubated with isotype controls in place of primary antibodies (not shown). Staining for FBG and EGF-L was observed in areas of fibrosis and around blood vessels, and largely overlapped (mean overlap coefficient = 0.94 (+/-SD 0.02), R = 0.645 (+/-SD 0.11)(n=5)(merged channels: bottom left panel). Areas of single reactivity for both EGF-L (green) and FBG (red) antibodies were also observed; white arrows highlight sites where anti-FBG staining predominated (merged channels: bottom right panels). Scale bars 100 μm.

**Methods**

**Antigen production**

DNA constructs for antigen expression were codon optimized for expression in mammalian cells, synthesised and sequence confirmed by Genscript (Piscataway, USA).

***CD4-His-tagged FBG variants and Fc-His-tagged human FBG for antibody discovery:*** The FBG domains of human, canine, rat and murine tenascin-C, and the FBG domain of human tenascin-R, were cloned into a mammalian expression vector which fuses the expressed domains with an N-terminal rat CD4 (domains 3 and 4) tag and a his tag [1] or a human IgG1 Fc and his tag [18]. Transfection of HEK293 cells was carried out as described previously [2] and antigens were affinity-purified by immobilized metal affinity chromatography using Ni-NTA agarose (Qiagen).

***Fc-His-tagged human FBG for solid phase binding and primary cell assays:*** The FBG domain from human tenascin-C was cloned into a human IgG1 Fc- and his-containing pQMCF-3 expression vector (Icosagen). After sequence verification, 1μg of expression vector was transfected into CHOEBNALT85 cells cultured for 48 h after which time G418 was added to select a plasmid containing cell population. For protein production the temperature was shifted to 30°C and cells fed for 8 days, after which time the cultures were centrifuged at 1000 g, for 30 min at 15°C, PMSF was added and the supernatants frozen until purification. For purification supernatants were thawed and filtered (0.22µm) and purification carried out using HiTrap MabSelect SuRe 5ml columns followed by gel filtration using HiLoad 16/600 Superdex 200pg columns into PBS pH 7.0.

***His-tagged human FBG for X-ray crystallography:*** The FBG domain from human tenascin-C was cloned with an N-terminal 6-his tag into a pQMCF-3 expression vector (Icosagen). After sequence verification, 1µg of expression vector was transfected into CHOEBNALT85 cells cultured for 48 h after which time G418 was added to select a plasmid containing cell population. For protein production the temperature was shifted to 30°C and cells fed for 8 days, after which time the cultures were centrifuged at 1000 g, for 30 min at 15°C, PMSF was added and the supernatants frozen until purification. For purification supernatants were thawed and filtered (0.22µm) and purification carried out using a 5ml HisTrap FF column followed by gel filtration (HiLoad 16/600 Superdex 200pg) into PBS pH7.0.

Protein purity and concentration was assessed by SDS-PAGE (4-12% gel) and spectrophotometry (OD280 using theoretical extinction coefficient). Endotoxin content was determined using the limulus amoebocyte lysate assay (Pierce) and proteins with endotoxin levels >1 EU/mg discarded. Table M1 summarizes the protein constructs made in this study and their applications.

**Table M1. Protein constructs synthesized for use in this study.** Boundary domains were based on those used for the FBG domain from human tenascin-C as we previously defined in [11]. CD4-his tagged FBG domains from human tenascin-C and –R were used in the primary antibody screen to select antibodies specific for tenascin-C that did not cross react with tenascin-R. CD4-his tagged FBG domains from canine, rat and murine tenascin-C were used in SPR assays to determine species cross reactivity. Fc-his tagged FBG from human tenascin-C was used to stimulate cells and in solid phase binding assays to de-select antibodies recognizing the CD4 tag and not the FBG antigen. His-tagged FBG from human tenascin-C was used in structural studies to reduce the risk posed by larger tags of interference with crystallization.

| **Antigen** | **DNA accession number** | **FBG domain amino acid coordinates** | **Tag** | **Annotation** | **Use** |
| --- | --- | --- | --- | --- | --- |
| **Human tenascin-C** | NM_002160.3 | 1974-2201 /2201 | Rat CD4 + his | CD4-his-hTNC-FBG | Primary screen, SPR, affinity maturation |
|  |  |  | Human IgG1 Fc + his | Fc-his-hTNC-FBG | HEK produced: primary screen, counter selection, cell line reporter assay screen |
|  |  |  |  |  | CHOEBNALT85 produced: primary cell activation, solid phase binding |
|  |  |  | His only | His-hTNC-FBG | Crystallization |
| **Canine tenascin-C** | NM_001195149.1 | 1974-2201 /2201 | Rat CD4 + his | CD4-his-cTNC-FBG | SPR |
| **Murine tenascin-C** | NM_011607.3 | 1792-2019 /2019 | Rat CD4 + his | CD4-his-mTNC-FBG | SPR, affinity maturation |
| **Rat tenascin-C** | NM_053861.1 | 1792-2019 /2019 | Rat CD4 + his | CD4-his-rTNC-FBG | SPR |
| **Human tenascin-R** | NM_003285.2 | 1128-1358 /1358 | Rat CD4 + his | CD4-his-rTNR-FBG | Primary screen, SPR, affinity maturation |

**Phage display selection and primary screening**

Selection of scFv binders from the IONTAS phage display library of 4 x 10^10^ clones and primary time resolved fluorescence (TRF) assay screening for FBG domain binders were performed as described previously [3]. Briefly, selected antibodies from 2 rounds of phage display were sub-cloned using *Nco* I and *Not* I restriction endonuclease sites into a vector for expression of soluble scFv [4] and transformed into *E. coli* strain BL21 (DE3). Individual scFv clones were picked, expressed in 96-well format, and scFv-containing supernatants tested for binding to immobilised CD4-his-hTNC-FBG (coated at 10 μg/ml) and lack of binding to CD4-his-hTNR-FBG (10 μg/ml) using the DELFIA (dissociation-enhanced lanthanide fluorescence immunoassay) system.

**Cell line reporter assay screen**

THP1-Blue^TM^ cells (Invivogen) carrying an NF-κB inducible secreted alkaline phosphatase (SEAP) reporter construct were added to a 96-well tissue culture plate (10^5^ cells/ well) and stimulated with Fc-his-hTNC-FBG (3 nM) in RPMI media (total volume 180 µl) in the presence of purified Fab antibodies (20 µl at 10x the indicated final concentration). Samples were incubated for 18 h at 37°C and SEAP activity was quantified using AttoPhos AP fluorescent substrate (Promega).

**BIAcore surface plasmon resonance (SPR) measurements**

SPR experiments were performed using a BIAcore T100 instrument using the Human antibody capture kit protocol (GE, BR-1008-39) according to the manufacturer’s instructions. Dissociation was measured over 10 minutes and experiments were performed at 37° unless otherwise stated. Kinetic parameters were determined by reference cell subtraction and fitting of the sensogram experimental data to a 1:1 interaction using the BIAevaluation software.

**Affinity maturation of anti-FBG antibodies**

Affinity maturation was performed by diversification of the CDR3 regions of NCS20 using oligonucleotide-directed (NNK) mutagenesis. Randomisation of contiguous blocks of 6 amino acid residues in the heavy and light chain CDR3s was carried out as described previously [5-7]. Stop codons were introduced to the phagemid templates (encoding the parental NSC20 scFv antibody) prior to library generation by Kunkel mutagenesis. The resulting phage libraries were subjected to stringent solution phase selection using biotinylated CD4-his-hTNC-FBG or CD4-His-mTNC-FBG and streptavidin Dynal beads as described previously [8]. Phage-antibodies were pre-incubated with soluble CD4-his-hTNR-FBG to “de-select” antibodies that also bind to the FBG domain of tenascin-R. The selected scFv populations were sub-cloned into the bacterial expression vector pSANG10 [9], transformed into *E. coli* BL21 (DE3), and individual transformants picked (46 per selection) for screening.

**HTRF and “expression-normalised capture” (ENC) DELFIA screens**

Samples and reagents for Homogeneous Time Resolved Fluorescence (HTRF) were prepared in assay buffer (50 mM NaPO4, 0.1% BSA, 0.4 M KF, pH 7.0). NCS20 IgG4 was labelled using the d2 labelling kit (CisBio, 62D2DPEA) as directed by the manufacturer and used at 1.25 nM. Biotinylated CD4-his-hTNC-FBG was prepared using EZ-link Sulfo-NHS-LC-Biotin reagent (Thermo Scientific, 21327) the extent of biotinylation was quantified using biotinylation fluorescence quantitation kit (Thermo Scientific, 46610). Biotinylated CD4-his-hTNC-FBG was pre-incubated with streptavidin europium cryptate for 45 minutes before adding to the (Greiner 384-well) assay plate at a final concentration of 1 nM. Supernatants containing scFv were diluted 1/20 in the final 20 μl reaction. Samples were incubated for 1 hour at room temperature and the FRET (fluorescence resonance energy transfer) signal was determined using a BMG Pherastar.

For the ENC assays black immunosorb plates (Nunc) were coated overnight (4°C) with anti-FLAG M2 antibody (Sigma, 5 μg/ml in PBS, 50 μl per well). After plate blocking and washing, auto-induction culture supernatants [10] containing expressed scFv were blocked in PBS-M (PBS, 3% Marvel) by mixing 1:1 with double strength buffer before adding to the assay plate (50 μl per well). Plates were washed three times with PBS-T (PBS, 0.1% Tween-20) and three times with PBS. Binding of biotinylated CD4-his-mTNC-FBG (5 μg/ml in PBS-M, 50 μl per well for 1 hour) was detected using Europium-labelled Strepravidin (Perkin Elmer, 1 μg/ml in PBS-M, 50 μl per well for 30 minutes). All conditions, reagents, wash steps and incubation times not explicitly stated were performed as described previously [3].

**Antibody production**

DNA sequences encoding the C3 heavy and light chain variable regions were cloned into an IgG4 S241P heavy / kappa light chain expression vector [11]. IgG4 and his-tagged Fab antibodies for screening and SPR analysis were produced by transient transfection of HEK293 cells as described previously [18]. Protein affinity purification employed either Ni-NTA agarose (Qiagen) or immobilised recombinant protein A resin (Generon) for Fab or IgG respectively. Antibody preparations were tested for purity and endotoxin as described above for antigen preparations. IgG4 antibodies for cell stimulation and in vivo models were produced by transfection of CHO-cells. The production culture was harvested (day 11) and clarified supernatant was concentrated by tangential flow filtration before being purified on a Mabselect SuRe column (GE Healthcare). Products were neutralised by the addition of 2x PBS, and adjusted with dilute sodium hydroxide solution to approximately pH 7.2 and stored in aliquots at 5°C.

DNA sequences encoding the heavy and light chain variable regions of NSC20 were cloned into a murine IgG2a heavy / kappa light pQMCF-3 expression vector (Icosagen). IgG2a antibodies for immunohistochemistry were produced by transfection of CHOEBNALT85 cells cultured for 48 h after which time G418 was added to select a plasmid containing cell population. For protein production the temperature was shifted to 30°C and cells fed for 9 days, after which time the cultures were centrifuged at 1000 g, for 30 min at 15°C, PMSF was added and the supernatants frozen until purification. For purification supernatants were thawed and filtered (0.22µm) and purification carried out using a 5ml Protein G affinity chromatography followed by Superdex 200 gel filtration into PBS, pH 7.4 and stored at -70°C.

**X-ray crystallography**

Crystallization was performed by Evotec Ltd. Fab’ of antibody C3 with protected cysteine residues was produced by pepsin cleavage (Thermo scientific), followed by incubation with 2-MEA (Thermo scientific) and treatment with NEM according to manufacturer’s instructions. The Fab’ was purified by size exclusion chromatography using a Superdex200 column. The Fab’-FBG complex was formed by incubating 20.6 mg Fab’ and 10mg hs-hTNC-FBG. The mixture was incubated on ice for 1 hour prior to purification by size exclusion chromatography, using a Superdex200 column. The FBG/Fab’ complex was crystallized in sitting drops at 22°C against a reservoir solution of 30 μl containing 100 mM Bis-tris pH 6.5, 200 mM magnesium chloride, 20% PEG 6000 in low profile Swiss-Sci 3 well plates. The crystals belong to the space group P1 2_1_ 1 with cell dimensions a=75.8 Å; b=116.9 Å; c=78.7 Å, and α=γ=90°; β=90.4°. Two molecules of the FBG/Fab’ complex are in the asymmetric unit. Crystals were cryo protected in 20% (v/v) glycerol (diluted in reservoir solution), prior to flash-cooling in liquid nitrogen. X-ray diffraction data was collected from a single crystal on a PILATUS 6M-F at the ID30B beamline station at the European Synchrotron Radiation Facility synchrotron, Grenoble, France. The wavelength of the X-ray beam was 0.9763 Ǻ. The data were processed with XDS and aimless [12, 13]. The crystal structure of human tenascin-C FBG domain/Fab’ complex was determined by Molecular Replacement using the program Phaser [14]. Modified versions of PDB entries 4R9J and 5I15 were used as starting models for Molecular Replacement. Remodelling, rebuilding and refinement was undertaken using COOT, Buccanner and Refmac respectively [15-17]. The final resolution was determined as 1.9 Å. The model of FBG / Fab’ complex encompasses residues 1975 to 2193 of tenascin-C (Uniprot: P24821), residues 1 to 213 of the light chain and residues 1 to 219 of the heavy chain. The R-factor of the model is 0.150, and R-free is 0.197. The r.m.s. deviation from standard geometry is 0.025 Å for bond lengths and 2.20° for bond angles.

**Solid phase binding assays**

Binding of proteins to the extracellular domain of TLR4 was carried out as described [18]. Briefly, GreinerBio medium-bind plates were coated with recombinant human TLR4 (R&D Systems) diluted to 1 µg/ml in PBS, or PBS alone, by overnight incubation at 4°C. After washing and blocking plates, a range of concentrations of Fc-his-hTNC-FBG were added and incubated for 2 h at room temperature, before washing again. Fc-his-FBG was detected with an anti-IgG1 antibody (BioRad) at 1 µg/ml, followed by incubation with sheep anti-rabbit HRP conjugated secondary antibody (AbD Serotec) and TMB substrate (KPL). Plates were read at 450 nm with a BMG FLUOstar OMEGA Microplate reader. For inhibition assays Fc-his-hTNC-FBG was pre-incubated with antibody C3 or NSC20 for 30 min at 37°C before addition to TLR4-coated plates and incubation at 37°C for 1 h.

**Primary human cell culture and stimulation**

Human monocytes were isolated from platelet apheresis residues (NHS Blood and Transplant, UK) using ficoll-gradient density separation and counter-flow centrifugation elutriation as previously described [16]. Monocytes were differentiated into macrophages by culture with M-CSF at 100 ng/ml (Peprotech) in RPMI 1640 medium (Lonza) supplemented with 5% heat inactivated FBS (Life technologies) for 5 days. Macrophages were plated at a density of 0.5x10^6^/ml in 96-well plates in RPMI 1640 medium supplemented with 3% heat inactivated FBS and 100 U/ml penicillin/streptomycin (Life technologies) and rested for 24 h before treatment with LPS at 1 ng/ml (Enzo) or Fc-his-hTNC-FBG at 1 µM. Stimuli were pre-incubated with anti-FBG or isotype control antibodies for 30 min at 37 ^o^C before addition to cells. Cell supernatants were collected after 24 h for analysis of cytokine levels by ELISA as described [8]. Cell viability was unaffected by all treatments as assessed by 3-(4,5-Dimethylthiazol-2-yl)-2,5-Diphenyltetrazolium Bromide (MTT) assays (Life technologies).

RA synovial membrane tissue was obtained at the time of joint replacement surgery/synovectomy with informed consent as described [19] and as approved by the local Trust ethics committee. A mixed population of all synovial cell types were isolated from the tissue using enzymatic digestion as previously described [17]. RA synovial membrane cells were plated at a density of 0.5x10^6^/ml in 96 well plates in RPMI 1640 medium supplemented with 10% heat inactivated FBS and 100 U/ml penicillin/streptomycin and with LPS at 1 ng/ml (Enzo) or Fc-his-hTNC-FBG at 1 µM. Stimuli were pre-incubated with anti-FBG or isotype control antibodies for 30 min at 37 ^o^C before addition to cells. Cell supernatants were collected after 24 h for analysis of cytokine levels by ELISA Cell viability was unaffected by all treatments as assessed by MTT assay.

**Immunofluorescence**

Synovial tissue from 28 patients in the Birmingham Beacon early arthritis cohort was analysed. In this cohort synovial tissue is obtained using ultrasound-guided biopsy [20]. Patients had clinically apparent synovitis of ≥1 joint, with inflammatory joint symptoms of ≤3 months duration, and had received no treatment for joint inflammation. Patients were followed for 18 months to ensure development of full disease phenotype. At 18 months patients were assigned to the following outcome categories: persistent RA according to ACR 2010 criteria [21] (RA, n=11), and resolving arthritis (no clinically apparent joint swelling, no DMARD/steroid use in the previous 3 months, n=9). Patients who presented at biopsy with symptoms of >3 months meeting ACR criteria were classified as established RA (n=8). Demographic and clinical parameters were recorded, and RA patients divided into anti-CCP antibody positive and negative subsets [22, 23]. All human samples were obtained with written, informed consent and approval from local medical ethical committees in compliance with the Declaration of Helsinki.

**Table M2. Details of arthritis patients used for immunostaining analysis**

Age and symptom duration are shown as median values with standard deviation in brackets. Rheumatoid factor (RHF) negative is ≤ 14IU/ml, anti CCP (Cyclic Citrullinated Peptide) negative is ≤6U/ml. *Data on NSAID and prednisone use prior to biopsy is not available for two established RA patients.

|  | **Sex** | **Age** | **Joint** | **Symptom duration/weeks** | **NSAID before biopsy?** | **Prednisone before biopsy?** | **Anti CCP negative** | **RHF negative** |
| --- | --- | --- | --- | --- | --- | --- | --- | --- |
| **Early RA** | 6/11 male | 59 (7.45) | 9/11 knee | 6 (3.2) | 6/11 | 1/11 | 9/11 | 11/11 |
| **Resolving arthritis** | 5/9 male | 40 (13.99) | 9/9 knee | 5 (2.3) | 6/9 | 0/9 | 9/9 | 9/9 |
| **Established RA** | 4/8 male | 59.5 (18) | 6/8 knee | 75.5 (62) | 3/6* | 0/6* | 1/8 | 1/8 |

Synovial biopsies embedded in OCT, frozen in liquid nitrogen vapour and stored at -80^o^C prior to sectioning. Sections (6μm) were cut and fixed in acetone for 20 minutes at 4^o^C. Prior to staining slides were re-hydrated in PBS and blocked in 10% normal horse serum PBS for 10 minutes. Primary antibodies were as follows: CD31 (mouse clone JC/70A or rat YR13), PDPN (clone NZ-1.3, eBioscience), and CD90 (clone F15-42-1, Millipore). The tenascin-C EGF-L domain was stained using clone 4F10TT from Takara/Clonetech; this antibody will detect full-length tenascin-C, as well as any fragments containing the EGF-L repeats, and the FBG domain using clone NSC20. Isotype controls were applied to sequential sections at the same concentrations. Following washing, secondary antibodies were applied from the following: goat anti IgG2a Alexa Fluor546 (Thermo), goat anti IgG2b AlexaFluor647 (Thermo), donkey anti rat Alexa Flour 647, goat anti IgG1 Alexa Fluor 488, and goat anti IgG1 FITC (Southern Biotech). After washing, if required, anti FITC Alexa Fluor 488 (Thermo) and Hoechst 33258 were added. Slides were washed and mounted with Prolong Diamond (Thermo). Sections were imaged on either the Zeiss Axio Scan.Z1 or Zeiss LSM880 confocal microscope prior to analysis. Zen blue software was used to determine the tissue area and the number of tenascin-C positive pixels (using isotype controls to control for background staining levels). Zen black software was used to determine co-localisation of both tenascin-C domains, using a derivation of the Manders method for colocalization and Pearson’s Correlation Coefficient [24].

**Rat CIA model**

Collagen-induced arthritis was performed by KWS, Bristol, UK. All experimental animal procedures were approved by the institutional ethics committee and the UK Home Office. Adult male Lewis rats were randomly allocated to experimental groups (10 animals per group) and allowed to acclimatise for one week. On day 0, animals were administered with 500 µl of a 1 mg/ml emulsion of type II bovine collagen in incomplete Freund’s adjuvant (CII/IFA) by intra-dermal injection in the lower back. On day 7, animals received a second injection of CII/IFA. Injections were performed under gas (isoflurane) anaesthesia. Antibody treatments were given on day 0, 3, 7, 10, 14, 17, 21 and 24 by IV injection. Treatment groups (each with 10 animals) were vehicle (PBS), control IgG4 10 mg/kg (Eureka Therapeutics), C3 1 mg/kg, C3 3 mg/kg, C3 10 mg/kg. From day 0 until the end of the experiment, animals were weighed three times per week. From day 7 until the end of the experiment, animals were scored three times per week for clinical signs of arthritis by an experimenter blind to the treatments. Each limb was scored on a five point scale: (0) absence of swelling, (1) slight swelling and/or erythema, (2) mild swelling, (3) moderate swelling and (4) severe swelling and/or joint rigidity and combined clinical scores from all 4 limbs was plotted. On Day 0, Day 14, Day 21 and Day 28, paw volumes were measured using a plethysmometer by an experimenter blind to the treatments. At termination, on Day 28, limbs were collected in tissue fixative for histopathology as described in [19]. Briefly, limbs were decalcified and processed for paraffin embedding. Sections were cut from both hind limbs of each rat (n=20 limbs per treatment group) and stained with haematoxylin and eosin (H&E). Sections were scored by an observer blind to the experimental design, for signs of arthritis to include inflammation, articular cartilage damage and damage to the underlying metaphyseal bone. Rats with any signs of histological disease were scored ‘Yes’, rats with no overt disease were scored ‘No’.

**Statistical analysis**

Statistical analysis was conducted using GraphPad Prism 7.02.

**Supplementary references**

1. Chapple, S.D., et al., *Multiplexed expression and screening for recombinant protein production in mammalian cells.* BMC Biotechnol, 2006. **6**: p. 49.

2. Falk, R., et al., *Generation of anti-Notch antibodies and their application in blocking Notch signalling in neural stem cells.* Methods, 2012. **58**(1): p. 69-78.

3. Schofield, D.J., et al., *Application of phage display to high throughput antibody generation and characterization.* Genome Biol, 2007. **8**(11): p. R254.

4. Martin, C.D., et al., *A simple vector system to improve performance and utilisation of recombinant antibodies.* BMC biotechnology, 2006. **6**: p. 46.

5. Fellouse, F., and Sidhu, SS *Making antibodies in bacteria. Making and Using Antibodies (Howard GC & Kaser MR, Eds.), CRC Press, Boca Raton, FL, pp. 157-180.* 2007.

6. Kunkel, T.A., J.D. Roberts, and R.A. Zakour, *Rapid and efficient site-specific mutagenesis without phenotypic selection.* Methods Enzymol, 1987. **154**: p. 367-82.

7. Sidhu, S.a.W., GA *Phage Display: A Practical Approach. Constructing phage display libraries by oligonucleotide-directed mutagenesis ,* , ed. H.a.C. Lowman, T; Oxford University Press, Oxford, UK, pp. 27–41. 2004.

8. Dyson, M.R., et al., *Mapping protein interactions by combining antibody affinity maturation and mass spectrometry.* Anal Biochem, 2011. **417**(1): p. 25-35.

9. Martin, C.D., et al., *A simple vector system to improve performance and utilisation of recombinant antibodies.* BMC Biotechnol, 2006. **6**: p. 46.

10. Studier, F.W., *Protein production by auto-induction in high density shaking cultures.* Protein Expr Purif, 2005. **41**(1): p. 207-34.

11. Angal, S., et al., *A single amino acid substitution abolishes the heterogeneity of chimeric mouse/human (IgG4) antibody.* Mol Immunol, 1993. **30**(1): p. 105-8.

12. Evans, P.R. and G.N. Murshudov, *How good are my data and what is the resolution?* Acta Crystallogr D Biol Crystallogr, 2013. **69**(Pt 7): p. 1204-14.

13. Kabsch, W., *Xds.* Acta Crystallogr D Biol Crystallogr, 2010. **66**(Pt 2): p. 125-32.

14. McCoy, A.J., et al., *Phaser crystallographic software.* J Appl Crystallogr, 2007. **40**(Pt 4): p. 658-674.

15. Cowtan, K., *The Buccaneer software for automated model building. 1. Tracing protein chains.* Acta Crystallogr D Biol Crystallogr, 2006. **62**(Pt 9): p. 1002-11.

16. Emsley, P., et al., *Features and development of Coot.* Acta Crystallogr D Biol Crystallogr, 2010. **66**(Pt 4): p. 486-501.

17. Murshudov, G.N., A.A. Vagin, and E.J. Dodson, *Refinement of macromolecular structures by the maximum-likelihood method.* Acta Crystallogr D Biol Crystallogr, 1997. **53**(Pt 3): p. 240-55.

18. Zuliani-Alvarez, L., et al., *Mapping tenascin-C interaction with toll-like receptor 4 reveals a new subset of endogenous inflammatory triggers.* Nat Commun, 2017. **8**(1): p. 1595.

19. Midwood, K., et al., *Tenascin-C is an endogenous activator of Toll-like receptor 4 that is essential for maintaining inflammation in arthritic joint disease.* Nat Med, 2009. **15**(7): p. 774-80.

20. Kelly, S., et al., *Ultrasound-guided synovial biopsy: a safe, well-tolerated and reliable technique for obtaining high-quality synovial tissue from both large and small joints in early arthritis patients.* Ann Rheum Dis, 2015. **74**(3): p. 611-7.

21. Aletaha, D., et al., *2010 rheumatoid arthritis classification criteria: an American College of Rheumatology/European League Against Rheumatism collaborative initiative.* Ann Rheum Dis, 2010. **69**(9): p. 1580-8.

22. Raza, K., et al., *Predictive value of antibodies to cyclic citrullinated peptide in patients with very early inflammatory arthritis.* J Rheumatol, 2005. **32**(2): p. 231-8.

23. Raza, K., et al., *Early rheumatoid arthritis is characterized by a distinct and transient synovial fluid cytokine profile of T cell and stromal cell origin.* Arthritis Res Ther, 2005. **7**(4): p. R784-95.

24. Manders, E., Verbeek, F., Aten, J., *Measurement of co-localization of objects in dual-colour confocal images* Journal of Microscopy, 1993. **169**(3): p. 375-382.
